# A Dispensatory, or Commentary on the Pharmacopœias of Great Britain (and the United States:) Comprising the Natural History, Description, Chemistry, Pharmacy, Actions, Uses, and Doses of the Articles of the Materia Medica

**Published:** 1848-09

**Authors:** 


*A Dispensatory, or Commentary on the Pharmacopœias of Great Britain (and the United States); comprising the Natural History, Description, Chemistry, Pharmacy, Actions, Uses, and Doses of the articles of the Materia Medica.* By ROBERT CHRISTISON, M. D., V. P. R. S. E., etc., etc. *Second Edition, revised and improved, with a Supplement, containing the most important new remedies, wth copious additions, and two hundred and thirteen illustrations.* By R. EGLESFELD GRIFFITH, M. D., Author of a Medical Botany, etc., etc. 8vo. pp. 1008. Lea & Blanchard. Philadelphia, 1848.

The first edition of Dr. Christison's Dispensatory was published at Edinburgh in 1842. Since then, the rapid strides of Chemistry have made us acquainted with improved modes of preparing and compounding some valuable medicines, and various articles have been added to our list of the Materia Medica, necessarily imposing on the author very extensive alterations of the text, as well as the addition of much important matter. These modifications and additions, so far as the wants of British practitioners are concerned, have been made by the distinguished author; but to adapt the work to the necessities of American pharmaceutists and prescribers, further additions were required, and these have been made with much care and accuracy by the editor.

